# Modeling ecological minimum requirements for distribution of greater sage-grouse leks: implications for population connectivity across their western range, U.S.A

**DOI:** 10.1002/ece3.557

**Published:** 2013-04-22

**Authors:** Steven T Knick, Steven E Hanser, Kristine L Preston

**Affiliations:** 1 U.S. Geological Survey, Forest and Rangeland Ecosystem Science Center970 Lusk Street, Boise, Idaho, 83706; 2 Center for Conservation Biology, University of California1303 Webber Hall, Riverside, California, 92521

**Keywords:** Ecological minimums, greater sage-grouse, landscape modeling, partitioned Mahalanobis *D*^2^, population connectivity, sagebrush, species distribution models

## Abstract

Greater sage-grouse *Centrocercus urophasianus* (Bonaparte) currently occupy approximately half of their historical distribution across western North America. Sage-grouse are a candidate for endangered species listing due to habitat and population fragmentation coupled with inadequate regulation to control development in critical areas. Conservation planning would benefit from accurate maps delineating required habitats and movement corridors. However, developing a species distribution model that incorporates the diversity of habitats used by sage-grouse across their widespread distribution has statistical and logistical challenges. We first identified the ecological minimums limiting sage-grouse, mapped similarity to the multivariate set of minimums, and delineated connectivity across a 920,000 km^2^ region. We partitioned a Mahalanobis *D*^2^ model of habitat use into *k* separate additive components each representing independent combinations of species–habitat relationships to identify the ecological minimums required by sage-grouse. We constructed the model from abiotic, land cover, and anthropogenic variables measured at leks (breeding) and surrounding areas within 5 km. We evaluated model partitions using a random subset of leks and historic locations and selected *D*^2^ (*k* = 10) for mapping a habitat similarity index (HSI). Finally, we delineated connectivity by converting the mapped HSI to a resistance surface. Sage-grouse required sagebrush-dominated landscapes containing minimal levels of human land use. Sage-grouse used relatively arid regions characterized by shallow slopes, even terrain, and low amounts of forest, grassland, and agriculture in the surrounding landscape. Most populations were interconnected although several outlying populations were isolated because of distance or lack of habitat corridors for exchange. Land management agencies currently are revising land-use plans and designating critical habitat to conserve sage-grouse and avoid endangered species listing. Our results identifying attributes important for delineating habitats or modeling connectivity will facilitate conservation and management of landscapes important for supporting current and future sage-grouse populations.

## Introduction

**Figure 1:**
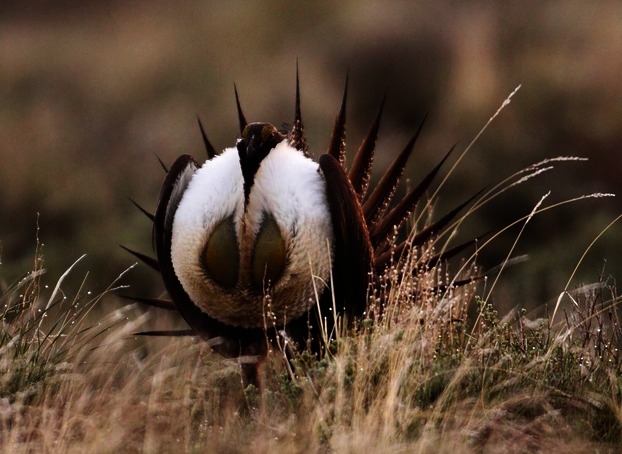
A male greater sage-grouse displays on a lek (traditional breeding ground). Photo credit: Matt T. Lee.

The greater sage-grouse *Centrocercus urophasianus* (Bonaparte) is an obligate resident of semiarid sagebrush *Artemisia* (L.) shrublands in western North America (Fig. 1). Although sage-grouse are still widely distributed across 11 states and 2 provinces, their current range is only 56% of their historical distribution prior to Euro-American settlement (Schroeder et al. 2004). Greater sage-grouse was recently designated as a candidate species for listing under the U.S. Endangered Species Act (U.S. Fish and Wildlife Service 2010). Although biological data coupled with lack of regulatory mechanisms warranted listing, endangered status was precluded because other species were considered to be higher priorities.

Sage-grouse are managed as an umbrella species for over 350 species of plants and animals that depend on sagebrush (Suring et al. 2005). The long-term future for this ecosystem is uncertain (Davies et al. 2011). Extensive regions of sagebrush have been burned by wildfire or lost to agriculture, energy and infrastructure development, and other resource demands by increasing human populations within the sage-grouse range (Knick et al. 2011). Remaining sagebrush landscapes are threatened further by exotic plant invasions leading to altered fire regimes and conversions to unsuitable expanses of exotic annual grasslands (Chambers et al. 2007; Miller et al. 2011; Balch et al. 2013). Long-term effects of changing climate could result in further loss of sagebrush by the end of this century: as much as 80% of the current sagebrush distribution could disappear under extreme projections (Neilson et al. 2005). Thus, current trajectories and future loss of sagebrush are likely to further imperil sage-grouse and other dependent species (U.S. Fish and Wildlife Service 2005, 2010).

Sage-grouse differ from many threatened species whose populations often are at risk because of localized ranges, restrictive habitat requirements, or are jeopardized by a dominant stressor. In contrast, sage-grouse are broadly distributed, occupy a diversity of environments containing sagebrush, and face multiple but cumulative threats throughout their range (Knick and Connelly 2011). Because conservation resources and time are limiting, delineating important areas and connecting corridors among populations could help focus actions in critical regions. Spatially explicit models delineating habitat for a species are important tools for directing land use or planning long-term conservation (Guisan and Zimmerman 2000; Elith et al. 2006). Numerous species distribution models have been developed for sage-grouse and have been important for understanding site-specific habitat relationships (Aldridge and Boyce 2007; Doherty et al. 2008; Shepherd et al. 2011). However, translating these habitat relationships into broad-scale maps has been hindered due to limited availability of accurate and consistent data spanning regional or range-wide distributions. Standard statistical approaches also present challenges because models based on ecological means, optimums, or correlational relationships often fail when applied to novel environments outside the inference space of the original data and do not accurately track either spatial or temporal change (Knick and Rotenberry 1998). Therefore, we used a partitioned Mahalanobis *D*^2^ model of resource selection to identify environmental characteristics that varied least at locations where a species occurs (Dunn and Duncan 2000; Browning et al. 2005). These consistent environmental characteristics, which correspond to an ecological niche, represent the most essential set of requirements limiting a species distribution (Rotenberry et al. 2002, 2006).

Identifying minimum requirements underlying sage-grouse distributions is particularly relevant because agencies responsible for managing sagebrush-dominated lands are considering sage-grouse needs while currently revising land-use plans and delineating priority regions (U.S. Bureau of Land Management 2011). Our second objective was to map a habitat similarity index (HSI) relative to the multivariate model of ecological minimums for the western portion of the sage-grouse range. We then converted the HSI to a resistance surface to model connectivity among delineated populations. These results are necessary to identify populations vulnerable to extirpation because of habitat loss or isolation, delineate potential corridors for movement among populations, and to provide a foundation from which to assess the implications of current or future habitat change.

## Study Area

**Figure 2:**
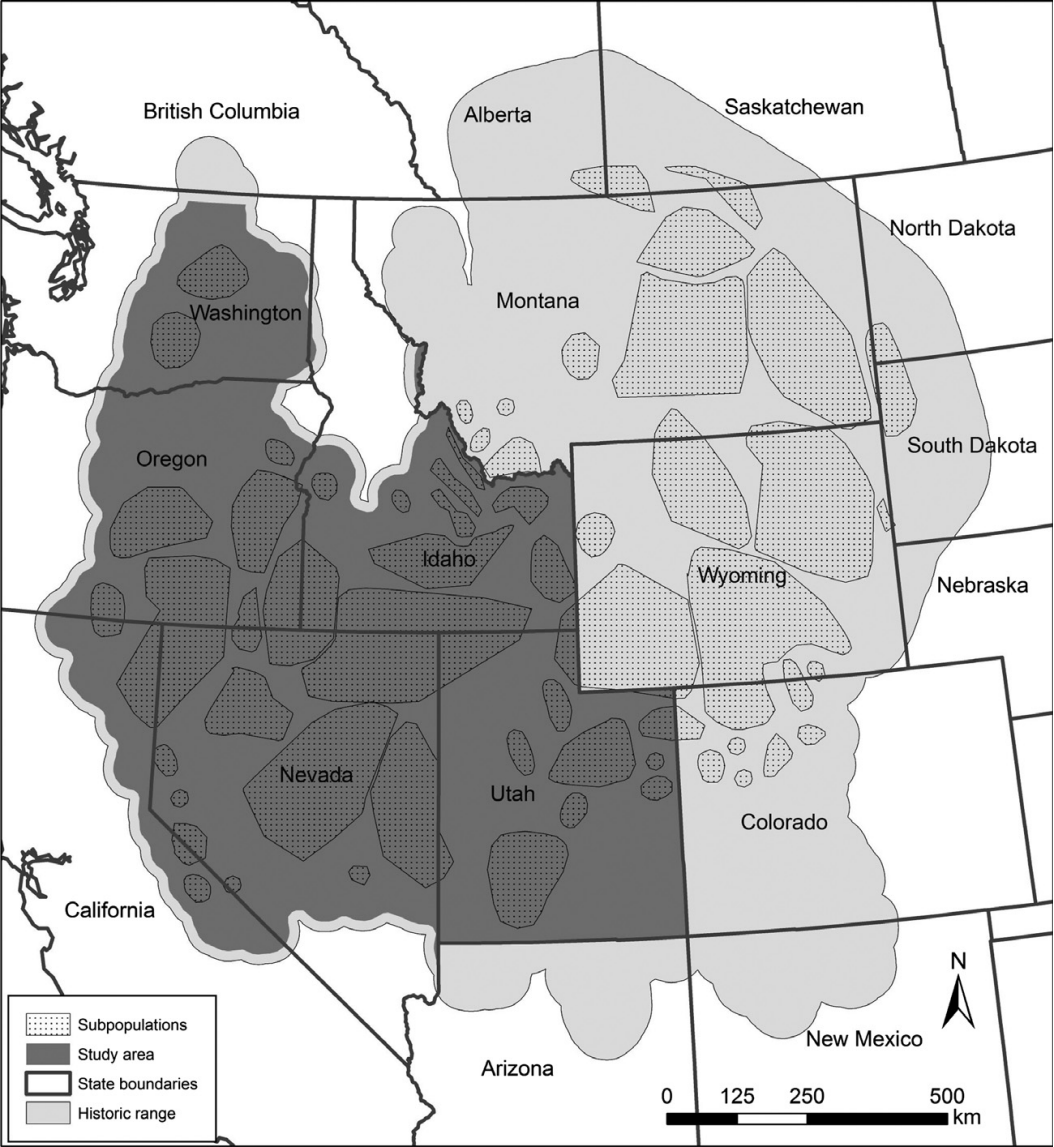
Study area and greater sage-grouse population boundaries within the historic sage-grouse range in western North America.

Our study area encompassed approximately 920,000 km^2^ of the western portion of the historic range occupied by sage-grouse, including areas outside of mapped population boundaries (Fig. 2) (Schroeder et al. 2004). A small part of our study area also included populations in the eastern range, which is generally delineated by the Rocky Mountains. The area is dominated by big sagebrush *A. tridentata* (Nutt.), little sagebrush *A. arbuscula* (Nutt.), and black sagebrush *A. nova* (A. Nelson) communities and is topographically and climatically diverse (Miller et al. 2011). Sage-grouse breed each spring (March–June) at traditional locations (leks) throughout this region. Thirty-six population units were delineated (Connelly et al. 2004), including six that may be extirpated based on absence of male sage-grouse at leks from 1998 to 2007.

## Materials and Methods

### Sage-grouse locations and environmental variables

We modeled species presence from locations of 3184 sage-grouse leks known to be active between 1998 and 2007. State wildlife biologists count displaying males each year to estimate population status; active leks were defined on an annual basis as those with ≥1 male sage-grouse attending (Garton et al. 2011).

We characterized the environment from land cover, anthropogenic, edaphic, topographic, and climatic variables represented in a 1-km grid within a Geographical Information System. We used an existing database of environmental variables that had been developed previously for broad-scale studies of sage-grouse population trend and habitat selection (Johnson et al. 2011; Wisdom et al. 2011). When possible, we matched time-specific predictor variables with the temporal period for lek data.

Most variables were measured for the 1-km grid cell within which the lek was located and also at larger scales represented by 5- and 18-km radii surrounding the lek location. We used these distances because a large proportion of females in nonmigratory and migratory populations nest within 5 and 18 km of the lek location (Connelly et al. 2000). Variables measured at 18-km radii did not perform as well in initial models as those at 5 km and were dropped in subsequent analyses.

The percentage of land cover class was measured from a 90-m resolution vegetation map (Landfire 2007). Land cover included agriculture, big sagebrush shrubland, big sagebrush steppe, conifer forest, developed, grassland, low sagebrush, mountain sagebrush, pinyon *Pinus* (L.) – juniper *Juniperus* (L.), riparian and all sagebrush types combined. Our environmental variables did not include understory components because these were not mapped explicitly (Landfire 2007). However, land cover communities described in the classification included associations for subdominant components.

We used fire perimeter data to characterize fire history by measuring total area burned between 1980 and 2007 (U.S. Geological Survey 2011a). Densities of anthropogenic features were developed from road, power line, pipeline, and communication tower distributions (U.S. Geological Survey 2011b). Soil variables were measured only at the lek location and included soil depth, available water capacity, salinity, and percent silt, clay, and sand (U.S. Department of Agriculture 2011). Topographic variables (slope and topographic heterogeneity) were calculated from a 90-m resolution raster-based digital elevation model (U.S. Geological Survey 2011c). We quantified local topographic heterogeneity using a vector ruggedness model (Sappington et al. 2007). Climate variables included mean annual, winter (November–February) and summer (May–August) precipitation, and mean annual minimum and maximum temperatures (Daly et al. 2004). Temperature and precipitation were averaged for 1998 through 2007 using 800-m resolution monthly climate data obtained from the PRISM Climate Group (Oregon State University 2011).

### Partitioned Mahalanobis *D*^2^

Mahalanobis *D*^2^ measures the standardized difference between the multivariate mean for *p* environmental variables calculated at *n* species occurrence locations and the values of those environmental variables at different points in the landscape being modeled (Clark et al. 1993). Smaller *D*^2^ values represent more similar conditions relative to the vector of multivariate means describing a species environment. An HSI can be created by rescaling *D*^2^ to range continuously from 0 to 1; an HSI of 1 indicates environmental conditions identical to the mean habitat vector whereas a value near 0 indicates very dissimilar conditions. Although these models identify areas most similar to characteristics of occupied habitat, other factors may determine actual occupancy (Pulliam 2000).

Mahalanobis *D*^2^ can be partitioned into *k* separate components, each reflecting independent relationships between a species occurrence and the set of selected environmental variables (Dunn and Duncan 2000; Rotenberry et al. 2002). Total number of partitions equals the number of variables in the model. Partitions are orthogonal and additive; summing all partitions equals the full rank model and provides the original *D*^2^ value. Independent partitions are derived in a principal components analysis (PCA) of the *n* × *p* matrix. An eigenvalue provides the variance accounted for by each partition and an eigenvector describes the linear contribution of each variable. Because partitions that have eigenvalues ≤1.0 explain little variance, they represent invariant environmental relationships in a species distribution. As such, these partitions define a multivariate model of limiting factors or environmental minimums (Dunn and Duncan 2000; Browning et al. 2005). Model precision can be increased by adding partitions, but at the cost of decreasing predictive capability.

### Model construction and evaluation

**Figure 3:**
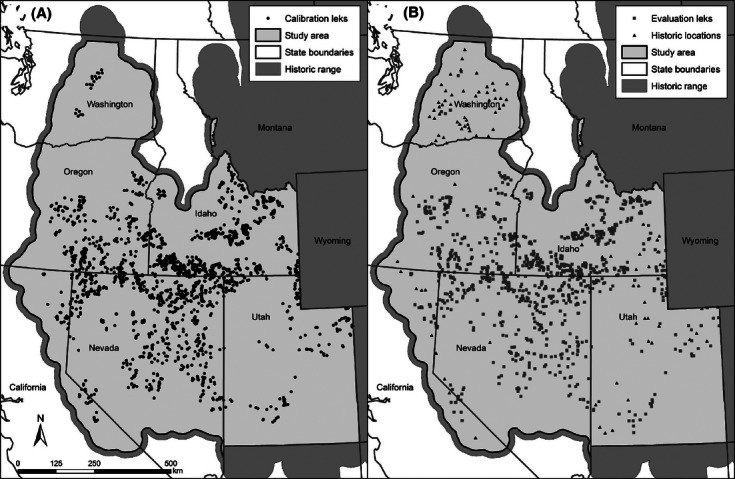
Distribution of greater sage-grouse lek locations active between 1998 and 2007 in the western range used to calibrate and evaluate models. Leks were randomly selected into calibration (A, black circles) and evaluation subsets (B, gray squares). Historic, but currently unoccupied sage-grouse locations (B, black triangles) were also used to test model performance.

We randomly selected 70% of the leks (*n* = 2070) to calibrate models (Fig. 3A) and withheld the remaining 30% (*n* = 1114) to evaluate performance (Fig. 3B). We estimated distributions of variables from 1000 iterative samples created by bootstrapping the calibration data. To better incorporate conditions in both large and small populations, we restricted the contribution from each population in a sample to a random selection of a maximum of 25 leks. We then performed a PCA on each of the 1000 iterative samples. The final model was created by subsequently averaging the PCA output after correcting for sign ambiguity (Bro et al. 2008) across all iterations.

We evaluated the ability of each *D*^2^(*k*) partition to predict habitat by calculating median HSI scores for calibration and evaluation data (Rotenberry et al. 2006). We also used 99 locations where sage-grouse historically occurred but are no longer extant to evaluate how well models distinguished current from unoccupied habitat (Wisdom et al. 2011). To further evaluate model performance, we calculated the area under the curve (AUC) for a receiver operating characteristic (ROC) to assess sensitivity (fraction of occurrences correctly classified) and specificity (fraction of unoccupied points predicted as occupied) (Fielding and Bell 1997). To calculate the AUC, we used the HSI values for 3184 randomly selected locations in the study area and for the 3184 lek to construct the ROC and calculate AUC (Phillips et al. 2006).

We used multiple criteria to select the final partition (Dunn and Duncan 2000). First, we examined each *k* partition having an eigenvalue ≤1.0 for relative differences in the spacing of eigenvalues among adjacent partitions. We also considered performance against evaluation data and our subjective knowledge of use areas predicted by each partition. Finally, we assessed the interpretability of eigenvector coefficients from the broader context of known sage-grouse biology (Connelly et al. 2011).

### Ecological minimums

We assumed first that all variables directly measured and included in the model contributed to the *p*-dimensional *D*^2^(*k*) space describing sage-grouse environmental requirements. We also assumed that variables not measured directly nonetheless were captured within that statistical characterization. We then identified variables that were highly correlated with partitions maintaining a consistent value where sage-grouse occurred (small eigenvalues <1). These variables were most likely to be associated with limiting factors compared to those correlated with partitions explaining large amounts of variation (larger eigenvalues) (Rotenberry et al. 2006). Finally, we considered a variable as an important contributor to the ecological minimum vector if it was correlated with the selected partition (eigenvectors > |0. 3| and to HSI scores (Halama et al. 2008).

We used dose-response curves (Hanser et al. 2011) to examine relationships between predicted HSI values and estimates for environmental variables measured at locations of sage-grouse leks active between 1998 and 2007 and for the study area grid. Relationships potentially identified include values for predictor variables relative to HSI scores at a threshold level estimated for 90% of the lek occurrences, strong linear relationships, or optimum of HSI scores. We also evaluated whether proportion of lek locations with high HSI scores differed from the proportion of points in the study area falling within that range of values. We calculated means and 95% confidence intervals for each variable to compare environmental characteristics among active leks, historic locations, and the study area.

### Population connectivity

We used mapped HSI scores to model pathways of potential sage-grouse movement among leks and populations (Circuitscape 3.5; McRae 2006). Models based on circuit theory treat landscapes as conductive surfaces to predict movement and connectivity patterns. Current flowing across the landscape can then be used to identify areas important for connectivity. Number, width, and permeability of available pathways determine the robustness of connections between two locations of interest (McRae et al. 2008). Important model attributes include strength of the current source, landscape resistance, and juxtaposition of current source to grounds. We set the strength of each current source equal to the mean annual count of individuals (1998–2007) at leks within 1-km cells to incorporate size variation. We assumed that individuals would move more easily through areas meeting their habitat requirements and estimated resistance for each 1-km cell in the study area by scaling the inverse of the HSI from 1 (low resistance/high HSI) to 100,000 (high resistance/low HSI). Areas outside the historic range of sage-grouse were given a value of 100,000 to reduce influence from map boundaries (Koen et al. 2010). Each lek cell was iteratively activated as a source with all others as ground that simulated an increased likelihood of individuals to move to adjacent leks. We combined all current (movement potential) map outputs to produce a cumulative map of connectivity.

## Results

Eighteen of 27 *D*^2^(*k*) partitions met our criteria of having an eigenvalue ≤1 (Table 1). We selected *D*^2^(*k* = 10) because of its relative difference among adjacent partitions (Δeigenvalue_*D*_^2^_(*k* = 9–10)_ = 0.10), performance against evaluation data (median HSI: evaluation leks = 0.85; historic locations = 0.0, AUC = 0.85), our subjective assessment of accuracy in map delineations (Fig. 3), and our ability to interpret *D*^2^(*k* = 10) based on relative importance of variables (Table 2).

**Table 1:** Model partition (*k*) and eigenvalues for a Mahalanobis *D*^2^ model of 27 environmental variables describing sage-grouse environments

Model partition (*k*)	Eigenvalue
1	3.85
2	2.98
3	2.36
4	1.85
5	1.70
6	1.48
7	1.29
8	1.18
9	1.11
10	1.01
11	0.94
12	0.86
13	0.81
14	0.75
15	0.67
16	0.56
17	0.53
18	0.49
19	0.46
20	0.43
21	0.40
22	0.32
23	0.29
24	0.23
25	0.21
26	0.13
27	0.11

Partition eigenvalues were averaged from 1000 models using iterative subsamples randomly drawn from 2070 active sage-grouse lek locations.

**Table 2:** Mean (SE), range, and absolute values of *D*^2^ (*k* = 10) eigenvectors for environmental variables measured at 3184 sage-grouse leks, 99 historic but currently extant locations, and for the study area

	Active leks		Historic		Study area		
				
Environmental variables	Mean (SE)	Range	Mean (SE)	Range	Mean (SE)	Range	Eigenvector *D*^2^ (*k* = 10)
Land cover (%)							
Big sagebrush shrubland	29.8 (0.4)	0–97.6	11.8 (1.3)	0–66.1	15.3 (0.02)	0–99.5	0.09
Big sagebrush shrub steppe	19.5 (0.4)	0–94.5	8.0 (1.1)	0–51.3	6.9 (0.01)	0–100	0.33
Low sagebrush	20.1 (0.4)	0–95.4	4.1 (0.9)	0–59.1	8.0 (0.01)	0–97.1	0.12
Mountain sagebrush	9.4 (0.3)	0–89.1	3.7 (1.1)	0–77.8	4.7 (0.01)	0–98.8	0.10
All sagebrush	78.84 (0.33)	1.93–99.98	34.87 (0.03)	0–100	27.7 (2.01)	0.43–80.22	
Agriculture	2.1 (0.1)	0–83.1	26.6 (2.4)	0–93.5	8.1 (0.02)	0–97.8	0.36
Conifer forest	0.8 (0.1)	0–44.4	3.4 (0.7)	0–40.6	12.5 (0.03)	0–99.1	0.21
Developed land	0.3 (0.01)	0–14.1	8.7 (1.5)	0–83.9	1.4 (0.004)	0–99.5	0.04
Grassland	2.2 (0.1)	0–71.0	9.8 (1.3)	0–61.2	3.8 (0.01)	0–84.1	0.09
Riparian	1.9 (0.1)	0–33.5	2.2 (0.5)	0–50.7	2.1 (0.003)	0–87.1	0.10
Burn							
Burned area 1980–2007 (ha)	1421 (40)	0–7974	587 (121)	0–6145	770 (2)	0–7974	0.18
Anthropogenic							
Secondary roads (km/km^2^)^1^	66.6 (0.6)	0–288.8	164.7 (16.5)	26.3–1242.6	75.7 (0.1)	0–1332.4	0.11
Highways (km/km^2^)^1^	2.0 (0.1)	0–32.3	11.0 (1.3)	0–58.7	3.4 (0.01)	0–77.1	0.12
Interstate highways (km/km^2^)^1^	0.1 (0.02)	0–19.8	3.8 (0.8)	0–46.6	0.6 (0.003)	0–52.0	0.33
Power lines (km/km^2^)^1^	2.5 (0.1)	0–34.6	14.4 (1.4)	0–52.1	4.3 (0.01)	0–79.5	0.11
Pipelines (km/km^2^)^1^	1.4 (0.1)	0–78.1	8.6 (1.5)	0–64.3	2.7 (0.01)	0–208.2	0.08
Communication towers (towers/km^2^)^1^	0.1 (0.01)	0–8.9	18.3 (5.5)	0–286.5	0.6 (0.01)	0–2005.3	0.22
Soil							
Soil depth (cm)	102.6 (0.7)	0–152.0	110.4 (4.1)	0–152.0	104.0 (0.1)	0–152.0	0.06
Sand (% soil volume)	28.8 (0.2)	0–85.5	32.0 (1.7)	0–90.2	30.5 (0.02)	0–92.0	0.14
Silt (% soil volume)	28.3 (0.2)	0–70.0	37.9 (1.7)	0–70.0	30.0 (0.02)	0–81.5	0.08
Clay (% soil volume)	21.5 (0.2)	0–50.1	14.8 (0.7)	0–34.5	15.8 (0.01)	0–57.4	0.34
Salinity (mmhos/cm)	1.1 (0.02)	0–10.7	0.9 (0.1)	0–11.0	1.6 (0.003)	0–21.1	0.16
Available water capacity (cm/cm)	4.2 (0.03)	0–12.3	5.6 (0.3)	0–12.3	4.7 (0.003)	0–25.0	0.04
Topography							
Slope (degrees)	3.1 (0.1)	0–26.0	5.7 (0.7)	0–36.0	7.3 (0.01)	0–69.3	0.15
Terrain ruggedness index	1.0 (0.1)	0–46.4	2.6 (0.7)	0–55.1	4.1 (0.01)	0–354.6	0.13
Climate							
Precipitation (mm)	333.3 (1.6)	169.0–835.8	329.3 (11.7)	140.4–782.0	376.3 (0.2)	76.4–3810.4	0.06
Minimum temperature (°C)	−9.5 (0.04)	−17.0 to −3.9	−6.6 (0.3)	−15.3 to −1.3	−8.3 (0.003)	−19.6 to 3.9	0.09
Maximum temperature (°C)	30.5 (0.03)	23.5–35.7	31.8 (0.2)	21.7–37.6	30.9 (0.004)	11.0–46.1	0.07

Land cover, burn area, and anthropogenic variables were measured within a 5-km radius of the lek. Soil, topography, and climate were measured at the lek location. Source data are available at http://sagemap.wr.usgs.gov.

1 Multiplied by 10^2^.

### Ecological minimums

**Figure 4:**
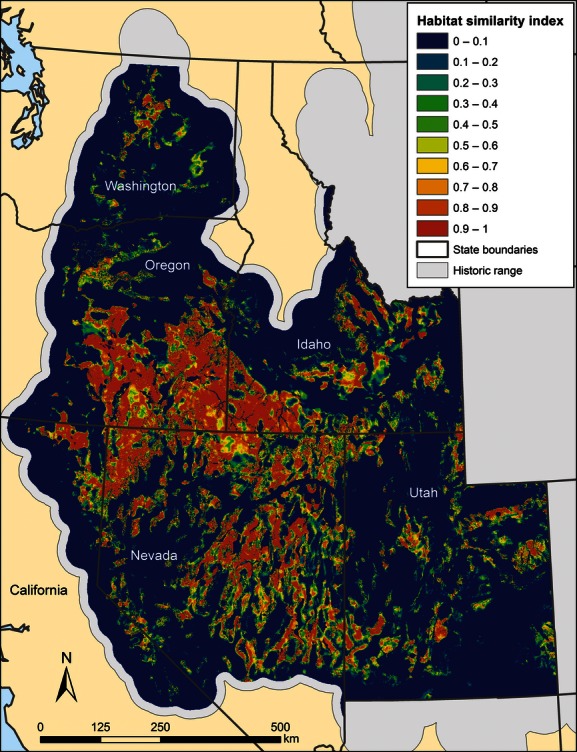
Habitat similarity index (HSI) values for greater sage-grouse across their western range. HSI values represent the relationship of environmental values at map locations to the multivariate model of minimum requirements for sage-grouse defined by land cover, anthropogenic variables, soil, topography, and climate.

Land cover of sagebrush and anthropogenic features were the primary variables defining the multivariate vector of ecological minimums (Table 2). Sagebrush in the surrounding landscape was highly important, particularly the big sagebrush shrub steppe type (Table 2). When all four sagebrush types were summed, 79% of the area within 5 km of the lek was in sagebrush land cover compared to 28% at 99 historic but no longer occupied locations and 35% for the study area. Lek locations had approximately twice the average large-scale sagebrush cover for the study area and nearly three times that of historic locations. Using the distribution of HSI scores for 90% of the leks as a threshold, active leks were surrounded by >40% landscape cover of sagebrush on average (Fig. 4A). Of the other dominant land cover types in our analysis, leks were absent from regions with ≥40% conifer and averaged <1% conifer forest within 5 km compared to an average of 13% for the study area and 3.4% for historic grouse locations (Table 2). Historic locations also had nearly five times more grassland and the study area nearly twice that of active leks (Table 2).

The HSI declined with increasing levels of human land use. Percent agriculture varied widely across individual lek locations, but <2% of the leks were in areas surrounded by >25% agriculture within a 5-km radius, and 93% by <10% agriculture (Fig. 4B). Ninety-nine percent of active leks were in landscapes with <3% developed; all lands surrounding leks were <14% developed (Fig. 4C). Historic locations where sage-grouse no longer occur were associated with landscapes dominated by >10 times the agriculture and >25 times the developed land as currently active leks (Table 2). Because large fires seldom occur in agriculture or developed landscapes, active leks had larger burned areas on average than historic locations and for the study area (Table 2).

Active leks also had lower densities of individual anthropogenic features than the study area or historic sage-grouse locations (Table 2). High lek HSI scores (≥0.60) were associated with large-scale densities of <1.0 km/km^2^ of secondary roads, 0.05 km/km^2^ of highways, and 0.01 km/km^2^ of interstate highways. Ninety-three percent of active leks fell below this threshold for interstate highways (Fig. 4D). Habitat suitability was highest at power line densities <0.06 km/km^2^ and pipeline and communication tower densities <0.01 km/km^2^. Leks were absent from areas where power line densities exceeded 0.20 km/km^2^, pipeline densities exceeded 0.47 km/km^2^, or communication towers exceeded 0.08 km/km^2^.

Active leks were situated on shallow slopes with less rugged terrain compared to the study area or historic locations (Table 2). No leks were characterized by slopes ≥27° or terrain ruggedness ≥0.05, although the study area included slopes to 70° and terrain ruggedness to 0.35. Mean annual precipitation for active leks and historic locations was on average 88% of that for the study area (Table 2) and varied from 169 to 835 mm. Minimum annual temperatures were lower at active leks and the study area compared with historic sage-grouse locations, whereas maximum annual temperatures were similar across datasets (Table 2). Maximum temperature varied between 11 and 46°C across the study area but was 27 to 32°C at leks having the highest HSI values.

### Population connectivity

**Figure 5:**
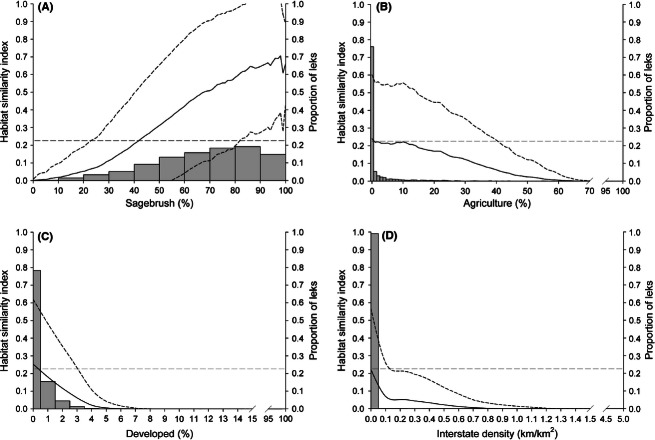
Changes in the habitat similarity index (HSI) relative to (A) sagebrush, (B) agriculture, (C) developed lands, and (D) density of interstate highways in the landscape within 5 km. Mean HSI values for study area (black line, ±1 SD [stippled lines]) and proportion of total leks (gray bars) were calculated for each increment of the environmental variables. Range of environmental variable values relates to the values within the study area. The dashed horizontal line indicates the HSI value (0.22) above which characterizes 90% of active leks.

**Figure 6:**
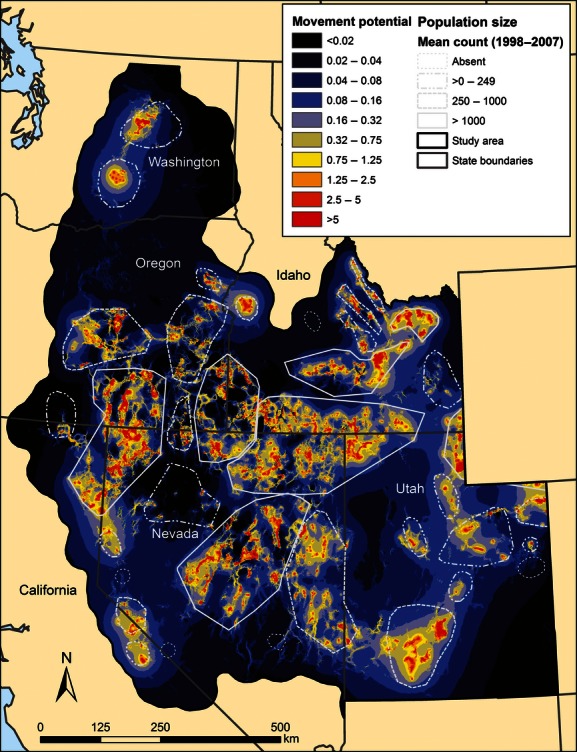
Estimated potential for sage-grouse movement among sage-grouse leks (Circuitscape; McRae 2006). Rescaled HSI values were used as a measure of landscape resistance.

The majority of populations were connected through landscapes characterized by moderate-to-high potential for animal movement (≥0.16, Fig. 5). Notable exceptions included both the Columbia Basin (Washington) and Bi-State (California–Nevada) Distinct Population Segments. Movement potential was higher among leks within individual populations than between populations. Large core populations in Nevada, Oregon, and Idaho were especially well connected. Small populations (mean annual count of males summed across all leks <250) were smaller in spatial area and had lower connectedness compared to large populations. Five populations with no active leks observed between 1998 and 2007 had limited connectivity to only one or two neighboring populations; four of these also were among the smallest designated populations by area (Fig. 6).

## Discussion

Sage-grouse are broadly distributed across western North America and occupy landscape matrices that vary widely in cover and configuration of sagebrush and other environmental characteristics (Johnson et al. 2011). Given this variability, it is difficult to accurately model habitat at fine spatial and thematic resolutions across the species range. Trade-offs are inherent because statistical relationships developed from small study extents can have high accuracy and use specific environmental variables, but have little predictive power elsewhere. Conversely, models developed from a general set of broad-scale, range-wide variables often fail to capture critical environmental factors specific to local areas (Scott et al. 2002). Therefore, developing a habitat model for sage-grouse required an approach that not only captured the spatial variability in their local environments but also maximized accuracy when applied across broad spatial extents. We developed and mapped an HSI representing a multivariate vector of ecological minimums that accurately discriminated the majority of lek locations including those in small, outlying populations from the study area and also from historic, but unoccupied locations.

### Ecological minimums

Species distribution models provide insights into how a species is linked to its environment. Alternative forms of statistical functions and models each address different questions relative to species-habitat relationships (Scott et al. 2002; Elith et al. 2006). Among these statistical options, partitioned *D*^2^ models that identify ecological minimums may not only be useful for modeling species distributions across large or changing environments but also provide important insights into that basic combination of factors necessary to support a species (Rotenberry et al. 2002; Browning et al. 2005). We used variables for land cover and human activities variables that affected sage-grouse directly but also included soil and abiotic characteristics because of their influence on distribution of sagebrush. We could not model fine-grained features, such as grass and forb understory composition, despite their seasonal importance to sage-grouse (Connelly et al. 2011) but suggest that these unmeasured components were captured within the environmental space of the ecological minimum.

Each partition of a *D*^2^ model delineates a relationship between a species and a multivariate configuration of the selected variables. We selected the partition that defined ecological minimums based on multiple but somewhat subjective criteria (Dunn and Duncan 2000). Of the partitions having eigenvalues <1.0, *D*^2^(*k* = 10) provided the best combination of ability to identify lek locations in independent evaluation data, accurately map current sage-grouse regions based on known distributions, and was readily interpreted relative to sage-grouse habitat requirements.

The multivariate vector defined by *D*^2^(*k* = 10) not only clearly reflected dependence on sagebrush by sage-grouse but also revealed other factors associated with core environmental conditions in landscapes used by sage-grouse. Minimum thresholds for sagebrush land cover required by sage-grouse in the landscape are emerging from this and other range-wide studies. In this study, 90% of the active leks had at least 40% of the large-scale landscape dominated by sagebrush, which compares to 25% to 30% sagebrush within 18- and 30-km scales previously identified as necessary to support sage-grouse persistence (Aldridge et al. 2008; Wisdom et al. 2011). Our estimate that 98% of the active leks were in regions containing <25% agriculture in the landscape also concurs with other range-wide analyses on effects of cultivated croplands (Aldridge et al. 2008; Wisdom et al. 2011). Leks were absent from areas with relatively low levels of anthropogenic development and infrastructure. Historic sage-grouse locations that currently are unoccupied were located in areas that now have high levels of development, indicating that human activity in addition to habitat loss may have contributed to extirpation from these areas (Aldridge et al. 2008; Wisdom et al. 2011). The ability of some leks to persist in landscapes containing lower amounts of sagebrush or greater levels of development likely was due to ameliorating presence of other ecological requirements.

Large-scale expansion and increasing dominance of invasive grasses in sagebrush shrublands at lower elevations is adversely affecting sage-grouse habitats (Knick et al. 2003). Synergistic feedbacks between invasive grasses and increased fire frequency and size has reduced sagebrush shrub cover and plant diversity and resulted in type conversions from sagebrush shrublands to non-native grassland landscapes (Davies 2011; Davies et al. 2011). The risk of further invasion by exotic grasses and ecosystem disruption over 100,000s of kilometers is moderate-to-high (Miller et al. 2011). At higher elevations, conifer and juniper woodlands are encroaching into sagebrush shrublands (Tausch et al. 1981; Miller et al. 2011), again resulting in lower habitat suitability for sage-grouse. Almost all leks were in areas containing little conifer or grassland cover in the surrounding landscape. Thus, two widespread trajectories of vegetation change are likely to further reduce habitat suitability across large areas of the sage-grouse range.

Active leks occurred only within a subset of the precipitation and temperature ranges even though climate varied widely across the study area. Sage-grouse currently occur in drier regions dominated by sagebrush. Thus, sage-grouse may have the ability to redistribute to areas that presently are cooler and wetter assuming that environmental conditions in new regions will be suitable and available for sagebrush expansion. The southwestern United States is projected to become more arid and is likely to experience more extensive and intensive droughts (Intergovernmental Panel on Climate Change 2007; Seager et al. 2007). Sage-grouse population extirpations have been linked to severe droughts (Aldridge et al. 2008), suggesting that populations in southern and more arid portions of the range may be most vulnerable.

### Population connectivity

Accurate maps of a species distribution are a primary goal of ecological niche-modeling (Elith et al. 2006). These maps can have an important role in conservation planning by delineating metapopulations and connecting corridors. Land and wildlife agencies currently are developing conservation actions for sage-grouse based on core or priority areas containing highest densities of breeding birds (Doherty et al. 2011). Less clear are land-use plans for regions outside of core areas that might be important for dispersal and gene flow. Species that have multiple interconnected populations are more likely to persist because risk of extirpation caused by regional events is confined to local populations; connectivity among populations ensures that recolonization can occur following local extirpation assuming that sufficient habitat remains (Thomas 1994; Hanski 1998). Populations within the interior portion of the sage-grouse range were highly interconnected. However, peripheral populations often were connected by habitat corridors only to one adjacent population. Human development or habitat loss that eliminates habitat in these corridors would further isolate those populations.

## Synthesis and Applications

Sagebrush shrublands are likely to be lost and fragmented in the future from a broad array of stressors (Miller et al. 2011). Extensive wildfires, expansion of agriculture, and development of utility and transportation infrastructures within the western range of the sage-grouse may continue to reduce habitat for sage-grouse across their western range. In addition, sagebrush distribution is predicted to decrease under future climate and land cover changes in the southern portion of the range may be most affected (Neilson et al. 2005; Bradley 2010). Leks persisting in landscapes already below the basic minimum ecological requirements might be most at risk and could be targeted for conservation actions. Minimum thresholds defining lek presence provide a basis from which to determine effects of projected or proposed levels of land use and anthropogenic development in areas that currently support active leks or to identify areas suitable for restoration of future sage-grouse habitat. We also caution that our results were based solely on lek locations. Although leks are important focal points for breeding and subsequent nesting in the surrounding region, other seasonal use areas and habitat requirements may be equally limiting to sage-grouse populations.

Population size and isolation can have serious negative impacts on genetic variability and population persistence (Frankham 2006; Höglund et al. 2007). Our mapped corridors of habitat among populations provide an important step in designing conservation actions that facilitate dispersal and gene flow and reduce isolation and risk of extirpation.
